# Transdiagnostic Predictors of Health-Related Quality of Life in Children with Autism and Epilepsy: A Cross-Sectional Study

**DOI:** 10.3390/jcm14020313

**Published:** 2025-01-07

**Authors:** Mirza Beg, Carly A. McMorris, Kim Smyth, Jeffery Buchhalter, Deborah Dewey

**Affiliations:** 1 Bachelor of Health Sciences Program, Cumming School of Medicine, University of Calgary, Calgary, AB T2N 4N1, Canada; mirza.beg2@ucalgary.ca; 2 Owerko Centre at the Alberta Children’s Hospital Research Institute, University of Calgary, Calgary, AB T2N 1N4, Canada; camcmorr@ucalgary.ca; 3 Werklund School of Education, University of Calgary, Calgary, AB T2N 1N4, Canada; 4 Department of Psychology, University of Calgary, Calgary, AB T2N 1N4, Canada; 5 Mathison Centre for Mental Health Research and Education, University of Calgary, Calgary, AB T2N 5A1, Canada; 6 Hotchkiss Brain Institute, University of Calgary, Calgary, AB T2N 4N1, Canada; kim.smyth@ahs.ca; 7 Department of Pediatrics, University of Calgary, Calgary, AB T3B 6A8, Canada; buchhalterj@gmail.com; 8 Department of Community Health Sciences, University of Calgary, Calgary, AB T2N 4Z6, Canada

**Keywords:** neurodivergent, health-related quality of life, transdiagnostic, autism, epilepsy, comorbid autism/epilepsy

## Abstract

**Background/Objectives**: Our understanding of the transdiagnostic factors that influence health-related quality of life (HRQOL) in individuals with neurodivergent conditions is very sparse and highly siloed by diagnosis labels. Research on transdiagnostic predictors of HRQOL across neurodevelopmental conditions is needed to enable care models that address shared needs of neurodivergent individuals beyond diagnostic boundaries. Our objective was to identify transdiagnostic factors associated with HRQOL in children with autism, epilepsy, or comorbid autism/epilepsy. **Methods**: This cross-sectional study included 37 autistic and/or epileptic children (mean age = 9.2; SD = 3.9; boys = 28). Parents provided sociodemographic information and completed the following measures: Social Communication Questionnaire (measure of severity of autistic symptoms); Parenting Stress Index, Fourth Edition; Pediatric Quality of Life Inventory; and the Behavioral Assessment System for Children, Third Edition. Child intellectual functioning was measured using age-appropriate scales: the Wechsler Preschool and Primary Scale of Intelligence-Fourth Edition: Canadian or the Wechsler Intelligence Scale for Children-Fifth Edition: Canadian. **Results**: Higher autistic symptom severity (OR = 0.851 95% CI: 0.732–0.988, *p* = 0.034) and parenting stress (OR = 0.687 95% CI: 0.493–0.959, *p* = 0.027) were associated with poorer HRQOL. Full Scale IQ and adaptive skills showed trend level associations with HRQOL. Sociodemographic factors including maternal education, child sex, and child age as well as child diagnosis were not associated with HRQOL. **Conclusions**: In this transdiagnostic sample of children, autism symptom severity and parenting stress were shared predictors of HRQOL. Interventions targeting child autistic symptoms and parents’ levels of stress could result in improved HRQOL in neurodivergent populations.

## 1. Introduction

Health-related quality of life (HRQOL) is a multidimensional concept that is used to examine the impact of health status on daily functioning and wellbeing [1]. HRQOL is typically evaluated through various indicators that reflect an individual’s health status and their physical and emotional functioning. It can be measured via self-report or reports from other relevant persons such as a caregiver, parent, or heath care professional. Research investigating HRQOL in children with neurodevelopmental conditions has examined children who have been diagnosed with specific conditions such as autism or epilepsy [2]. However, few studies have examined HRQOL transdiagnostically across neurodevelopmental conditions that frequently co-occur, such as autism and epilepsy; 12.1% of autistic people have a diagnosis of epilepsy and 9% of people with epilepsy have autism [3]. A transdiagnostic approach could be beneficial as it allows for the identification of shared challenges and strengths across conditions, thus leading to more flexible models of care.

Autism affects approximately one in 66 Canadian children [4] and is characterized by difficulties in social communication and social interactions, and the presence of restrictive and repetitive behaviors [5,6]. Autistic symptoms can range from mild to severe, and can be accompanied by learning problems, sleep problems, and cognitive delay [5,6]. Childhood epilepsy is a seizure disorder that is the result of abnormal electrical brain activity [7] and occurs in approximately 0.5% of children in Canada [8]. It is characterized by its episodic nature and can be associated with problems in behavior, sleep, and cognition. Neonatal- and infantile-onset epilepsies include Dravet Syndrome and epileptic spasms syndrome, which are characterized by developmental impairments including autism, have been linked to superimposed epileptic activity [9]. Childhood-onset epilepsies include syndromes such as Lennox–Gastaut Syndrome, epilepsy with myoclonic absences, and childhood absence epilepsy, which are often associated with comorbidities including autism [9]. A population-based case-control study in children with epilepsy found that children who have early onset of epilepsy (i.e., within the first year) had a higher risk of developing autism [10].

In children with autism and epilepsy, internalizing and externalizing behaviours and adaptive skills have been associated with HRQOL [11,12,13,14,15,16,17,18]. In some studies but not others, sociodemographic factors such as socioeconomic status, age, and gender have also been linked with HRQOL in children with autism and epilepsy [16,18,19,20,21]. Studies that have examined the association between IQ and HRQOL in autistic individuals have reported conflicting findings, with some reporting that IQ is associated with HRQOL while others reporting that it is not [13,15,16]. In contrast, IQ has consistently been associated with HRQOL in children with epilepsy [17,22]. In children with autism, more severe autistic symptoms have been associated with poorer HRQOL [12,19]; however, no studies have examined this association in children with epilepsy. Further, the influence of parenting stress on HRQOL has not been explored in children with autism or epilepsy. However, prior research has shown that mothers’ emotional states, including stress or depressive symptoms, can influence children’s well-being and relationships in various cultural settings [23,24]. Mothers of children with autism have been found to report higher levels of stress compared to mothers of typically developing children [25]. Considering that caregivers play an essential role in helping children with autism and/or epilepsy function in daily life, it is important that we understand the role of parenting stress in relation to HRQOL for children with these disorders [23,24]. A better understanding of the influence of child behaviour, adaptive skills, social communication, and IQ, and parenting stress on HRQOL could contribute to the development of interventions that may well result in improved HRQOL of children with autism, epilepsy, or comorbid autism and epilepsy.

There is a growing concern that existing discrete diagnostic categories do not align with underlying biological mechanisms, effectively guide the selection of interventions, or adequately capture the experiences of individuals with neurodivergent conditions. Therefore, a transdiagnostic approach may provide us with a better understanding of HRQOL in these individuals [2]. Neurodevelopmental conditions like autism and epilepsy are highly heterogeneous in terms of the cognitive and behavioural characteristics of individuals with these diagnoses [2]. However, there can be significant overlap among different diagnostic conditions in terms of behavioural and mental health comorbidities, sleep difficulties, and challenges in communication and adaptive functioning [2]. These shared characteristics can affect HRQOL, making it essential to study the predictors of HRQOL across diagnostic boundaries [2]. Moreover, by identifying predictors of HRQOL that are shared across different diagnoses, it becomes possible to create care models that address the needs of individuals with various diagnoses rather than only focusing on one diagnostic condition at a time [2]. To our knowledge, no study has investigated factors associated with HRQOL in a transdiagnostic group of children with autism, epilepsy, or comorbid autism and epilepsy. Our primary objective was to identify transdiagnostic factors associated with HRQOL in children with autism, epilepsy, or comorbid autism/epilepsy. The study focused on children with autism and epilepsy and comorbid autism/epilepsy in order to capture the unique and overlapping challenges across these neurodivergent children, enabling a deeper understanding of the individual and combined impacts of these neurodevelopmental conditions on HRQOL. Secondary aims of this study were: (1) to investigate if HRQOL differed among autistic, epileptic or comorbid autistic/epileptic children; and (2) to explore if factors associated with HRQOL differed among these diagnosis groups. 

## 2. Materials and Methods

### 2.1. Study Design and Participant Recruitment

This was a cross-sectional study. Families were recruited through the Epilepsy and Autism Developmental Clinics at Alberta Children’s Hospital, Calgary, Canada. Interested parents were asked to contact the investigators via phone or email. A member of the research team then screened families to ensure that their children met the inclusion criteria. The inclusion criteria were: (1) the child had a confirmed diagnosis of autism and/or epilepsy by a medical professional with appropriate expertise; and (2) the child was 4 to 16 years of age. This age range was chosen as the standardized questionnaires and measures used in the study were validated for children across this age range. Forty-three parents and their children who met our inclusion criteria were enrolled into the study; of these, 37 were included in the final analyses. After enrollment some parents and their children chose not to participate and did not complete the questionnaires or the neurodevelopmental assessment. No specific reasons were provided. Therefore, they were not included in the final analyses. Of the families who chose to participate, the parents completed questionnaires that asked about family demographics, child behavior, social communication/autism severity, parenting stress, and child HRQOL. Permission to use these published standardized questionnaires was obtained from the publishers. The children participated in a neurodevelopmental assessment of intelligence. This project was approved by the Conjoint Health Research Ethics Board at the University of Calgary (REB-16-0472). Parents gave informed consent for themselves and their children, with the children providing assent.

### 2.2. Outcome Measure

The *Pediatric Quality of Life Inventory (PedsQL)* Parent Report measures health-related quality of life in healthy children and adolescents and those with acute and chronic health conditions and has established reliability and validity [26,27,28]. The PedsQL Parent Report contains 23 items covering four broad domains of physical and psychological health. These domains are physical functioning, emotional functioning, social functioning, and school functioning. A total scale score, which represents overall HRQOL, is computed from the scores of each of the four domains with a higher score indicating higher HRQOL. The PedsQL has been used in studies examining HRQOL in children with ASD and epilepsy [29,30]. It is able to evaluate both parental and child self-perceptions of HRQOL. Previous research has shown minimal correlation between parent-proxy and child self-reports, with parent-proxy reports generally reporting lower HRQOL levels than child self-reports [19,29]. The PedsQL has good psychometric properties with one study reporting a Cronbach’s alpha of 0.86 for the parent report [27]. PedsQL scores were dichotomized based on the established cut-off of 65.4 with higher scores indicating better HRQOL [31].

### 2.3. Predictors

The *Social Communication Questionnaire (SCQ)* was used to assess autistic symptom severity [32]. It includes 40 yes-no questions about social communication skills and behaviours and is completed by caregivers. The SCQ provides an indication of the approximate severity of autism in an individual. Scores range from 0 to 39 with higher scores indicating greater problems. A score of 12 is accepted as the cut-off point indicating the need for further assessment for autism [33]. The SCQ has been shown to have a sensitivity of 0.92 and a specificity of 0.62 in a sample of children with intellectual disability and with or without Pervasive Developmental Disorders [34]. It has a Cronbach’s alpha of 0.80 [35].

The *Behavioral Assessment System for Children—3rd Edition (BASC-3), Parent Report* has established reliability and validity in assessing children’s emotions and behaviors [36,37]. It contains 139–175 items depending on the version used, which is based on child age. For this study, three composite indexes were examined: Internalizing Problems, Externalizing Problems, and Adaptive Skills. Each index yields a T-score that ranges from 20 to 120. The mean T-score on the BASC-3 is 50 (SD = 10). Higher scores on Internalizing Problems and Externalizing Problems indicate more problems, with scores between 60–69 identified as “at risk” and scores over 70 indicating clinically significant problem behaviors. Lower scores on the Adaptive Skills index indicate poorer skills, with T-scores 30 to 39 considered “at risk” and scores below 29 indicating clinically significant problems. For children aged 4 to 16, BASC-3 Cronbach’s alphas for Externalizing Problems range from 0.91 to 0.96 For children aged 4 to 16, for Internalizing Problems, Cronbach’s alphas range from 0.92 to 0.96, and for Adaptive Skills they range from 0.94 to 0.97 [37].

*The Wechsler Preschool and Primary Scale of Intelligence—Fourth Edition, Canadian (WPPSI-IV^CND^) and Wechsler Intelligence Scale for Children—Fifth-Edition, Canadian (WISC-V^CND^)* were used to assess intelligence in children aged 4 years to 7 years, and 8 years to 16 years, respectively [38,39]. The WPPSI-IV^CND^ and WISC-V^CND^ provide a measure of Full-Scale IQ (FSIQ) derived from 5 indexes: Verbal Comprehension, Visual Spatial, Fluid Reasoning, Working Memory, and Processing Speed. The average standard score for Full-Scale IQ (FSIQ) and for each of the indexes is 100 (SD = 15). Higher scores indicate better performance. Both measures have excellent reliability and validity [40,41]. The WPPSI-IV^CND^ and WISC-V^CND^ have Cronbach’s alphas of 0.95 and 0.96, respectively [38,39].

The *Parenting Stress Index–Fourth Edition (PSI-4)* is a 120 item parent report questionnaire designed to investigate the magnitude of stress in the parent–child system and has established reliability and validity [42,43]. A raw total score ranging from 131 to 320 is attained by combining the Child and Parent domain scores. The Child domain examines sources of stress related to child characteristics, whereas the Parent domain investigates sources of stress associated with parent characteristics. There is also a Life Stress domain that includes 19 dichotomous questions examining the personal experiences of the parent in the last 12 months. A Total T-score is attained by combining the raw scores from the Child and Parent domain scores. The mean Total T-score on the PSI-4 is 50 (SD = 10) with higher scores indicate more stress. Scores at the 90th percentile or higher are considered clinically significant. The Cronbach’s alpha for the Total T-score is 0.98 [43].

### 2.4. Covariates

Potential covariates were selected based on previous studies that reported associations with HRQOL in children [16,18,19,20,21,44,45,46]. The covariates identified were sex (male, female), language spoken at home (English, not English), child race (White, non-White), parental marital status (married, not married), maternal education (high school or technical training, university degree or above), and annual household income (i.e., <CND 70,000, >CND 70,000). As English is the primary language spoken in Calgary, Canada, we chose to dichotomize language into English versus non-English for language spoken at home. Most participants were white, with only a few non-white participants; therefore, ethnicity was dichotomized as White vs. non-White. As Calgary has one of the most highly educated populations in Canada, we chose to compare those who did not have a university degree to those who had a university degree. Further, as 70K CND was the median income in Calgary at the time the study was conducted, it was used as the cut-off for income.

### 2.5. Statistical Analyses

Sociodemographic characteristics and clinical variables were examined using means (SD) or percentages, as appropriate. The Shapiro–Wilk test was conducted to assess the normality of the continuous variables. Results indicated that the Internalizing, Externalizing, and Adaptive Skills T-scores on the BASC-3 were normally distributed as evidenced by their Shapiro–Wilk test statistics of 0.90, 0.93, 0.83, respectively. Univariate logistic regressions were used to examine the association between each potential covariate and HRQOL. Consistent with previous research, covariates associated with HRQOL at *p* < 0.20 in the univariate analyses were used to adjust the logistic regressions for each of the predictors [47,48,49]. To address our secondary aims, Fisher exact tests and one-way analysis of variance tests (ANOVA) were used as appropriate to explore differences in demographic characteristics and predictors among the three diagnosis groups (autism, epilepsy, comorbid autism/epilepsy), with follow-up pairwise analysis carried out using Bonferroni correction if the ANOVA yielded a *p* < 0.05. An ANOVA was also used to investigate differences in HRQOL scores among the three diagnosis groups. We then conducted logistic regressions stratified by diagnosis group; however, no covariates were included due to the small sample sizes. This is an exploratory study; therefore, no power calculations were conducted. All analyses were carried out using STATA 18.5.

## 3. Results

Of the participants, 16 (43%) children were diagnosed with autism, 10 (27%) with epilepsy, and 11 (30%) with comorbid autism and epilepsy. The mean age of the children was 9.2 (SD = 3.0) years (range, 4 to 16 years), with six children under 6 years, 24 between 6 to 11 years, five between 12 to 14 years, and two who were 15 years or older. Most of the participants were male (n = 28); however, this differed among the diagnostic groups with higher proportions of males in the autism (i.e., 14/16) and comorbid autism/epilepsy (i.e., 9/11) groups and an equal proportion of males and females in the epilepsy group (five males, five females). Approximately two-thirds of the children were White (63.9%), and most families reported an annual income of more than 70,000 CND (64.7%). No significant differences in demographic characteristics were found among the three diagnostic groups; however, differences were noted in child Adaptive Skills and FSIQ (see Table 1).

The mean SCQ score in our neurodivergent sample was 11.6 (SD = 7.4), which is just below the proposed cutoff score of 12 for an autism diagnosis [33]. When examined by diagnostic group, the mean SCQ score for children in the epilepsy group was 7.5 (SD = 5.6); for children in the autism or comorbid autism/epilepsy the mean scores were 13.7 (SD = 6.9) and 12.1 (SD = 8.6), respectively. The mean Internalizing, Externalizing, and Adaptive Skills T-scores on the BASC-3 were 59.7 (SD = 14.0), 59.2 (SD = 11.3), and 31.9 (SD = 12.4), respectively, suggesting that the participants in this study were at risk for problems in Adaptive Skills. The mean FSIQ of the participants was 77.8 (SD = 21.9), which is below the mean FSIQ of the general population, which is 100 (SD = 15) [40,41]. The mean PSI-4 Total T-score was 58.1 (SD = 9.1), which is in the normal range. Finally, the mean score on the parent-proxy reports of the PedsQL was 62.2 (SD = 18.9). This is lower than the mean PedsQL parent-proxy report score reported for children 2 to 16 years of age without autism and/or epilepsy during initial field testing of the PedsQL [26]. The scores of the children without these conditions ranged from 78.59 (SD = 16.53) to 88.14 (SD = 12.11) depending on the age group observed [26]. After the PedsQL was dichotomized, 15 children had a score of 65.4 or above.

The results of the univariate logistic regression analyses for each covariate are presented in Table 2. Both marital status (OR = 7.000 95% CI: 0.705–69.490, *p* = 0.097) and child ethnicity (OR = 0.364 95% CI: 0.081–1.641, *p* = 0.188) were associated with child HRQOL at a *p*-value < 0.20 and therefore included as covariates in the analyses. Adjusted logistic regressions revealed that SCQ scores (OR = 0.851 95% CI: 0.732–0.988, *p* = 0.034) and PSI-4 scores (OR = 0.687 95% CI: 0.493–0.959, *p* = 0.027) were significantly associated with HRQOL (Table 3). Trend level associations were noted for FSIQ (OR = 1.041 95% CI: 0.993–1.091, *p* = 0.094) and BASC-3 Adaptive Skills (OR = 1.085 95% CI: 0.986–1.193, *p* = 0.095).

### Secondary Analyses: Associations Among Diagnostic Groups

No significant differences were found among diagnostic groups in HRQOL, *F*(2,29) = 0.66, *p* = 0.53 (Table 1). One-way analysis of variance (ANOVA) revealed a significant difference in Adaptive Skills among the three diagnosis groups: autism (*M* = 31.0, *SD* = 12.6), epilepsy (*M* = 39.3, *SD* = 13.4) and comorbid autism and epilepsy (*M* = 26.0, *SD* = 7.5), F(2, 32) = 3.33, *p =* 0.047. The effect size was large, with η^2^ = 0.172; however, these differences did not remain significant in post hoc pairwise analyses using Bonferroni adjustment (alpha 0.05/3 = 0.15) for multiple comparisons. One-way ANOVA revealed significant differences in FSIQ among participants diagnosed with autism (*M* = 82.6, *SD* = 20.7), epilepsy (*M* = 87.6, *SD* = 16.0), and comorbid autism and epilepsy (*M* = 60.7, *SD* = 20.5), *F* (2,29) = 5.05, *p* = 0.013. The effect size was moderate, with η^2^ = 0.258. Post hoc pairwise comparisons using Bonferroni correction with an alpha of (0.05/3 = 0.15) were not significant. Logistic regression analyses stratified by diagnostic group examining the associations of the SCQ, BASC-3 Adaptive Skills and Externalizing and Internalizing Problems scores, FSIQ, and PSI-4 with HRQOL did not reveal any significant associations (Tables S1–S3).

## 4. Discussion

This study investigated factors associated with HRQOL in a neurodivergent sample of children with autism and/or epilepsy. We found that higher autism symptom severity and higher parenting stress were significantly associated with poorer HRQOL. Children’s adaptive skills and FSIQ showed trend level associations with HRQOL. Internalizing and externalizing problems, diagnostic group, and demographic factors were not associated with poorer HRQOL.

Our finding that higher autism symptom severity was associated with lower HRQOL in our neurodivergent sample is consistent with previous research conducted with autistic children [11,12,13,14,15]. The SCQ includes measures of behaviors associated with autism and higher SCQ scores reflect more severe autistic behaviors, which could negatively impact quality of life. Increased autism symptom severity can result in greater challenges with verbal and non-verbal communication that can lead to misunderstandings that make understanding social cues, participating in conversations, and forming friendships harder, leading to social isolation and lower HRQOL [50]. Higher parenting stress was also associated with lower HRQOL. To the best of our knowledge no previous studies have examined associations between parenting/caregiver stress and child HRQOL in a neurodivergent sample of children with autism and/or epilepsy Our findings suggest that parenting stress may be an important predictor of HRQOL in children in neurodivergent populations and that further research is needed that investigates if interventions that reduce parenting stress in these populations results in improved HRQOL for these children and their families.

Adaptive skills were associated with poorer HRQOL at a trend level. Previous research has reported that poorer adaptive skills are associated with worst HRQOL [13,16,17,19]. Adaptive skills play a role in an individual’s independent functioning including communication, socialization, self-care, and motor skills. Underdeveloped adaptive skills can lead to challenges in navigating everyday life, maintaining social relationships, and succeeding academically or professionally resulting in reduced HRQOL [51].

Conflicting evidence has been reported on the association between FSIQ and HRQOL [13,15,16,17,22,52]. Howlin et al. found that FSIQ has a threshold effect on life outcomes as defined in terms of social, communication, and behavioral functioning, including measures like independence, employment status, and living situation [52]. Children with an IQ above 70 (high IQ) had significantly better life outcomes compared to those with an IQ below 70 (low IQ) [52]. Reilly et al. reported a similar trend in children with epilepsy [22]. Therefore, the lack of a significant association in the present study could be because the range of FSIQs among our sample may not have been broad enough.

Unlike some previous studies, we found no associations between age and gender and HRQOL [16,17,19]. Furthermore, we found no relationships between any other sociodemographic variables including family income, marital status, and maternal education and HRQOL. This could be because our neurodivergent population was very homogenous, with most participants coming from high-income, well-educated, and intact families. As a result, we may have lacked the needed variance to detect associations with HRQOL. Unlike previous research in autism and epilepsy populations, internalizing problems such as anxiety and depression were not associated with HRQOL [14,16,18,53]. Individuals with autism and/or epilepsy may display a wide range of symptoms and only severe internalizing problems may be associated with HRQOL. As the mean internalizing problems T-score of the participants in the present study was in the normal range (M = 59.7), this may have limited our ability to detect an association. Consistent with research investigating externalizing problems in children with epilepsy [18], we did not find that externalizing problems were predictive of HRQOL; however, this is contradictory to what has been reported in research with autistic children [16].

To our knowledge, no studies have compared the HRQOL of children with autism, epilepsy, or comorbid autism and epilepsy. We found that diagnosis was not associated with HRQOL, which suggests a high degree of variability in HRQOL among individuals within the same diagnostic category. Children with autism, epilepsy, or comorbid autism-epilepsy can have vastly different abilities and experiences, which could influence parent perceptions of their child’s HRQOL, and overshadow general trends associated with diagnosis. Importantly, these findings highlight that when considering HRQOL in populations with neurodevelopmental concerns, diagnostic label may not be a relevant factor. The findings of the present study support a transdiagnostic approach for assessing child HRQOL in neurodivergent populations.

Children who are neurodivergent, and particularly children with autism and epilepsy, as well as their families, often face widespread stigma and other societal barriers that negatively impact their HRQOL. Therefore, there is a need for research to understand factors that impact HRQOL and how these factors can be addressed to ensure the best possible outcomes. The focus of this study on identifying shared predictors across diagnostic categories aligns strongly with calls for addressing the interconnected needs of neurodivergent individuals and provides some initial groundwork for rethinking existing care models [2]. By highlighting shared predictors of HRQOL among neurodivergent children with differing diagnoses, the findings of this study provide some initial information that could inform public policy regarding resource allocation for tackling the most important predictors of HRQOL in these populations.

This study has notable strengths. It investigated a neurodivergent population of children with autism, epilepsy, and comorbid autism and epilepsy who displayed similar health and neurodevelopmental concerns, allowing us to enhance our understanding of the transdiagnostic factors associated with HRQOL across these different diagnostic conditions. It is the first study to examine the influence of parenting stress on HRQOL in these populations. Moreover, this study collected data on sociodemographic factors, which allowed us to investigate their relationships with HRQOL. There are also some limitations that should be acknowledged. The associations observed in the analyses should not be interpreted as causal due to the study’s cross-sectional design, which limits the ability to establish temporal or causal relationships. We used parent-proxy reports to assess HRQOL as many of the children in our sample were unable to complete a self-report measure on HRQOL. Using parent-proxy reports to assess child HRQOL comes with key considerations as parent-proxy and child self-reports often have low correlations; therefore, they should be treated as separate sources of information [11,19,54]. In future work, the inclusion of both parent-proxy and child self-reports should be considered. Also, this study did not consider factors such as religion, spirituality, and cultural background, which may impact parents’ perceptions of their child’s quality of life [55]. Another limitation of our study was its small sample size, which limits statistical power, increasing the risk of Type I and II errors. Although we examined the association between autism symptom severity and HRQOL, the association between severity of epilepsy symptoms and HRQOL was not examined. To address this gap in the literature, future research needs to consider epilepsy severity in relation to HRQOL. Finally, generalization of the results may be limited due to the homogeneity of the sample. Studies with larger and more diverse samples are required to support the findings of this study.

## 5. Conclusions

The findings of this study suggest that across a neurodivergent population of children with autism and/or epilepsy there may be shared transdiagnostic predictors of HRQOL including parenting stress and autistic symptoms. Further research on transdiagnostic predictors across neurodivergent populations is critically needed to enable care and interventions beyond diagnostic boundaries and to improve our understanding of HRQOL from the perspective of neurodivergent individuals, their families, and their communities. Given the small sample size and exploratory nature of this cross-sectional study, the findings should be interpreted cautiously.

## Figures and Tables

**Table 1:** Sociodemographic characteristics, predictors, and outcomes of the overall neurodivergent sample of children with autism and/or epilepsy, and by diagnostic group with *p*-values provided that indicate differences among diagnostic groups.

	Neurodivergent	Autism	Epilepsy	Autism and Epilepsy	Differences Among Diagnostic Groups *p*-Value	% Missing Data
**Sociodemographic Variables**						
Male; *n* (%)	28 (75.7)	14 (87.5)	5 (50.0)	9 (81.8)	0.13 *	0.0
English Spoken at Home; *n* (%)	30 (83.3)	11 (68.8)	10 (100)	9 (90)	0.13 *	2.7
White; *n* (%)	23 (63.9)	10 (62.5)	7 (70)	6 (60)	1.00 *	2.7
Income above CAD 70 000; *n* (%)	22 (64.7)	11 (68.8)	6 (66.7)	5 (45.5%	0.90 *	8.1
Child Age; *M* (*SD*)	9.2 (2.9)	8.3 (2.2)	8.7 (3.3)	10.8 (3.0)	0.07 **	0
Education past high school; *n* (%)	19 (55.9)	8 (53.3)	5 (55.6)	6 (60.0)	1.00 *	8.1
Married; *n* (%)	28 (80%)	12 (80.0)	7 (70.0)	9 (90)	0.51 *	5.4
**Predictors**						
Social Communication Questionnaire (SCQ); *M* (*SD*)	11.6 (7.4)	13.7 (6.9)	7.5 (5.6)	12.1 (8.6)	0.17 **	18.9
BASC-3 Internalizing Problems T-score; *M* (*SD*)	59.7 (14.0)	59.1 (16.5)	61.6 (12.8)	59.0 (12.3)	0.90 **	5.4
BASC-3 Externalizing Problems T-score; *M* (*SD*)	59.2 (11.3)	58.1 (13.1)	59.8 (11.8)	60.3 (8.6)	0.88 **	5.4
BASC-3 Adaptive Skills T-score; *M* (*SD*)	31.9 (12.4)	31.0 (12.6)	39.3 (13.4)	26.0 (7.5)	0.048 **	5.4
Full Scale IQ *M* (*SD*)	77.8 (21.9)	82.6 (20.7)	87.6 (16.0)	60.7 (20.5)	0.01 **	13.5
Parenting Stress Index-4 (PSI-4) T-Score; *M* (*SD*)	58.1 (9.1)	59.2 (9.5)	52.8 (6.7)	63.3 (8.1)	0.07 **	18.9
**Outcome**						
Pediatric Quality of Life Inventory (PedsQL) above 65.4; *n* (%)	15 (46.9)	6 (40.0)	5 (62.5)	4 (44.4)	0.59 *	13.5

* Fisher exact test; ** One-way ANOVA; *M*, Mean; *SD*, Standard Deviation; *n*, number of participants; BASC-3, Behavioral Assessment System for Children, Third Edition.

**Table 2:** Univariate logistic regression models of associations between potential covariates, and HRQOL measured by the PedsQL.

Potential Covariates	Odds Ratio (95% CI)	*p*-Value
Sex	0.369 (0.060–2.274)	0.283
Child Age	1.031 (0.803–1.324)	0.808
Language Spoken at Home	0.462 (0.071–2.994)	0.418
Child Ethnicity	0.364 (0.081–1.641)	0.188
Marital Status	7.000 (0.705–69.490)	0.097
Maternal Education	0.375 (0.083–1.693)	0.202
Income	0.500 (0.095–2.628)	0.413

CI, Confidence Interval.

**Table 3:** Logistic regressions of associations between predictors, and HRQOL measured by PedsQL adjusted for marital status and child ethnicity.

Predictors	Odds Ratio (95% CI)	*p*-Value
Full Scale IQ	1.041 (0.993–1.091)	0.094
BASC-3 Externalizing Problems	0.945 (0.857–1.041)	0.249
BASC-3 Internalizing Problems	0.950 (0.877–1.029)	0.210
BASC-3 Adaptive Skills	1.085 (0.986–1.193)	0.095
Social Communication Questionnaire	0.851 (0.732–0.988)	0.034
Parenting Stress Index 4	0.687 (0.493–0.959)	0.027

CI, Confidence Interval; BASC-3, Behavioral Assessment System for Children, Third Edition.

## Data Availability

The data that support the findings of this study are available from the corresponding author upon reasonable request.

## Supplementary Materials

The following supporting information can be downloaded at: https://www.mdpi.com/article/10.3390/jcm14020313/s1. Table S1: Logistic regression models of the associations between predictor variables and PedsQL in autistic children (n = 16); Table S2: Logistic regression models of the associations between predictor variables and PedsQL in children with epilepsy (n = 10); Table S3: Logistic regression models of the associations between predictor variables and PedsQL in children with comorbid autism and epilepsy (n = 11).
