# Supplementary material for: Transdiagnostic Predictors of Health-Related Quality of Life in Children with Autism and Epilepsy: A Cross-Sectional Study
Source: J Clin Med. 2025 Jan 7;14(2):313. doi: 10.3390/jcm14020313 (PMC11765855; doi:10.3390/jcm14020313)
Supplement: Supplementary file 1 [file jcm-14-00313-s001.zip › jcm-3359592-supplementary.pdf]

SUPPLEMENTARY MATERIALS

Transdiagnostic Predictors of Health-Related Quality of Life in Children with Autism and Epilepsy: A Cross-Sectional Study

Mirza Beg [1,2], Carly A. McMorris [2,3,4,5,6], Kim Smyth [6,7], Jeffery Buchhalter [7] and Deborah Dewey [2,6,7,8,*]

1 Bachelor of Health Sciences Program, Cumming School of Medicine, University of Calgary, Calgary, AB T2N 4N1, Canada; mirza.beg2@ucalgary.ca
2 Owerko Centre at the Alberta Children's Hospital Research Institute, University of Calgary, Calgary, AB T2N 1N4, Canada; camcmorr@ucalgary.ca
3 Werklund School of Education, University of Calgary, Calgary, AB T2N 1N4, Canada
4 Department of Psychology, University of Calgary, Calgary, AB T2N 1N4, Canada
5 Mathison Centre for Mental Health Research and Education, University of Calgary, Calgary, AB T2N 5A1, Canada
6 Hotchkiss Brain Institute, University of Calgary, Calgary, AB T2N 4N1, Canada; kim.smyth@ahs.ca
7 Department of Pediatrics, University of Calgary, Calgary, AB T3B 6A8, Canada; buchhalterj@gmail.com
8 Department of Community Health Sciences, University of Calgary, Calgary, AB T2N 4Z6, Canada
* Correspondence: dmdewey@ucalgary.ca

Table S1: Logistic regression models of the associations between predictors and PedsQL in autistic children (n = 16)

**Predictors**	**Odds Ratio (95% CI)**	***p*-value**
Full Scale IQ	1.014 (0.954-1.078)	0.652
BASC-3 Externalizing Problems	0.976 (0.890-1.069)	0.597
BASC-3 Internalizing Problems	0.893 (0.766-1.040)	0.146
BASC-3 Adaptive Skills	1.053 (0.960-1.154)	0.276
Social Communication Questionnaire	0.802 (0.617-1.044)	0.101
Parenting Stress Index-Fourth Edition	0.770 (0.588-1.007)	0.056

CI, Confidence Interval; BASC-3, Behavioral Assessment System for Children-3rd Edition.

Table S2: Logistic regression models of the associations between predictors and PedsQL in children with epilepsy (n = 10)

Predictors	Odds Ratio (95% CI)	p-Value
Full Scale IQ	1.165 (0.888-1.528)	0.271
BASC-3 Externalizing Problems	0.746 (0.459-1.214)	0.238
BASC-3 Internalizing Problems	0.939 (0.804-1.097)	0.429
BASC-3 Adaptive Skills	1.034 (0.912-1.172)	0.605
Social Communication Questionnaire	0.783 (0.503-1.220)	0.280
Parenting Stress Index-Fourth Edition	NA	Data perfectly predicted

CI, Confidence Interval; BASC-3, Behavioral Assessment System for Children-3rd Edition.

Table S3: Logistic regression models of the associations between predictor variables and PedsQL in children with comorbid autism and epilepsy (n = 11)

Predictors	Odds Ratio (95% CI)	p-Value
Full Scale IQ	1.061 (0.965-1.166)	0.224
BASC-3 Externalizing Problems	0.950 (0.788-1.146)	0.594
BASC-3 Internalizing Problems	0.991 (0.885-1.110)	0.875
BASC-3 Adaptive Skills	0.961 (0.612-1.509)	0.864
Social Communication Questionnaire	0.919 (0.768-1.099)	0.354
Parenting Stress Index-Fourth Edition	NA	Data perfectly predicted

CI, Confidence Interval; BASC-3, Behavioral Assessment System for Children-3rd Edition.
